# Supplementary material for: Sample Preparation and Warping Accuracy for Correlative Multimodal Imaging in the Mouse Olfactory Bulb Using 2-Photon, Synchrotron X-Ray and Volume Electron Microscopy
Source: Front Cell Dev Biol. 2022 Jun 8;10:880696. doi: 10.3389/fcell.2022.880696 (PMC9213878; doi:10.3389/fcell.2022.880696)
Supplement: Supplementary file 4 [file DataSheet1.pdf]

Sample preparation and warping accuracy for correlative multimodal imaging in the mouse olfactory bulb using 2-photon, synchrotron X-ray and volume electron microscopy

Yuxin Zhang[1, 2], Tobias Ackels[1, 2], Alexandra Pacureanu[1, 2, 3], Marie-Christine Zdora[4, 5, 6, 7], Anne Bonnin[7], Andreas T. Schaefer[1, 2]*, Carles Bosch[1]*

1 Sensory Circuits and Neurotechnology Lab., The Francis Crick Institute, London, UK
2 Department of Neuroscience, Physiology and Pharmacology, University College London, UK
3 ESRF, The European Synchrotron, Grenoble, France
4 Department of Physics and Astronomy, University College London, London, UK
5 Diamond Light Source, Harwell Science and Innovation Campus, Didcot, UK
6 School of Physics and Astronomy, University of Southampton, Highfield Campus
Southampton, UK
7 Paul Scherrer Institut, Villigen, Switzerland

Supplementary Figures and Tables

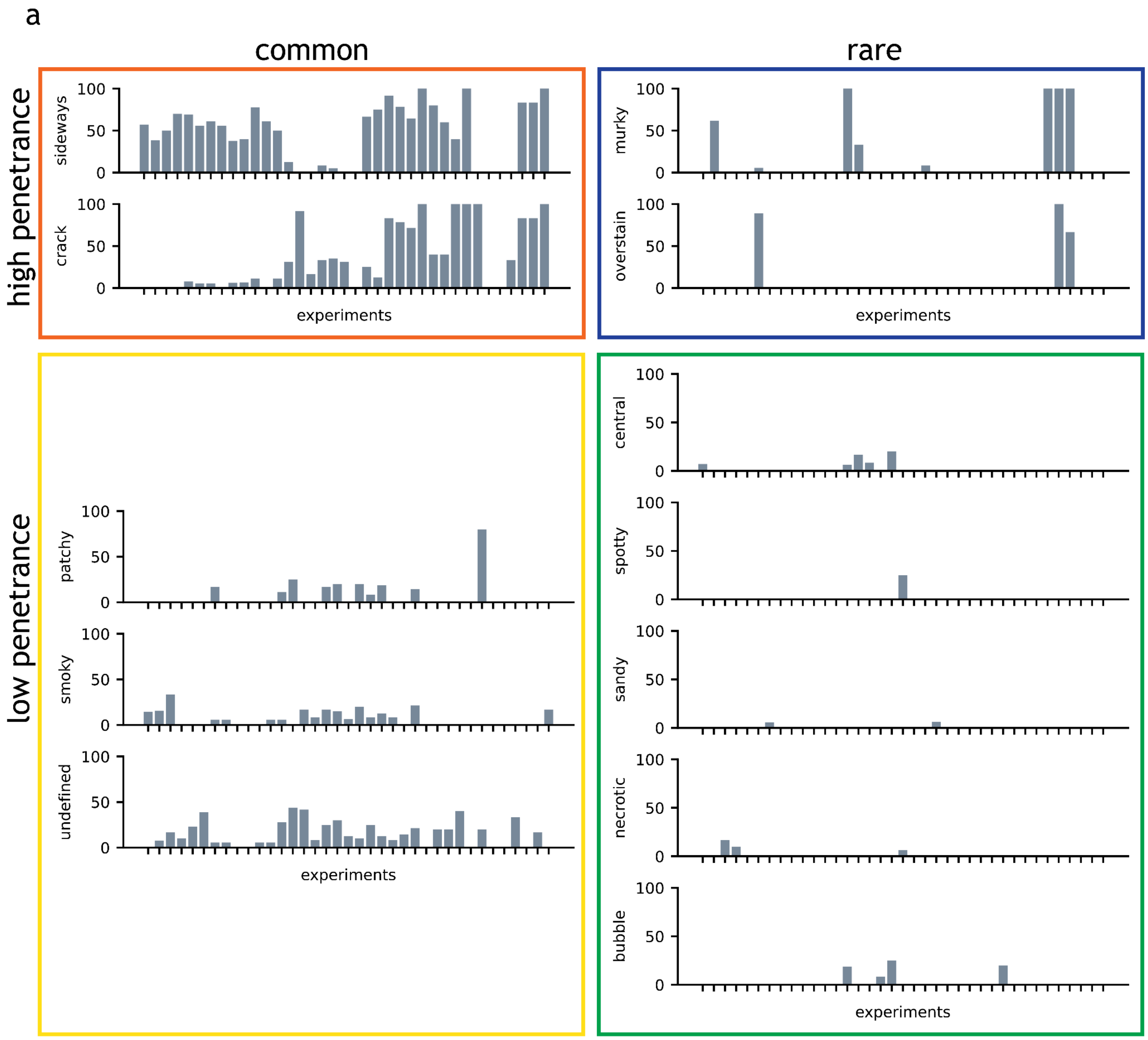

Supp. F1. Prevalence and penetrance of artefacts.

(a) Artefacts grouped by prevalence and penetrance. Prevalence describes the number of experiments containing samples with an artefact. Penetrance describes the percentage of samples affected by an artefact within one experiment.

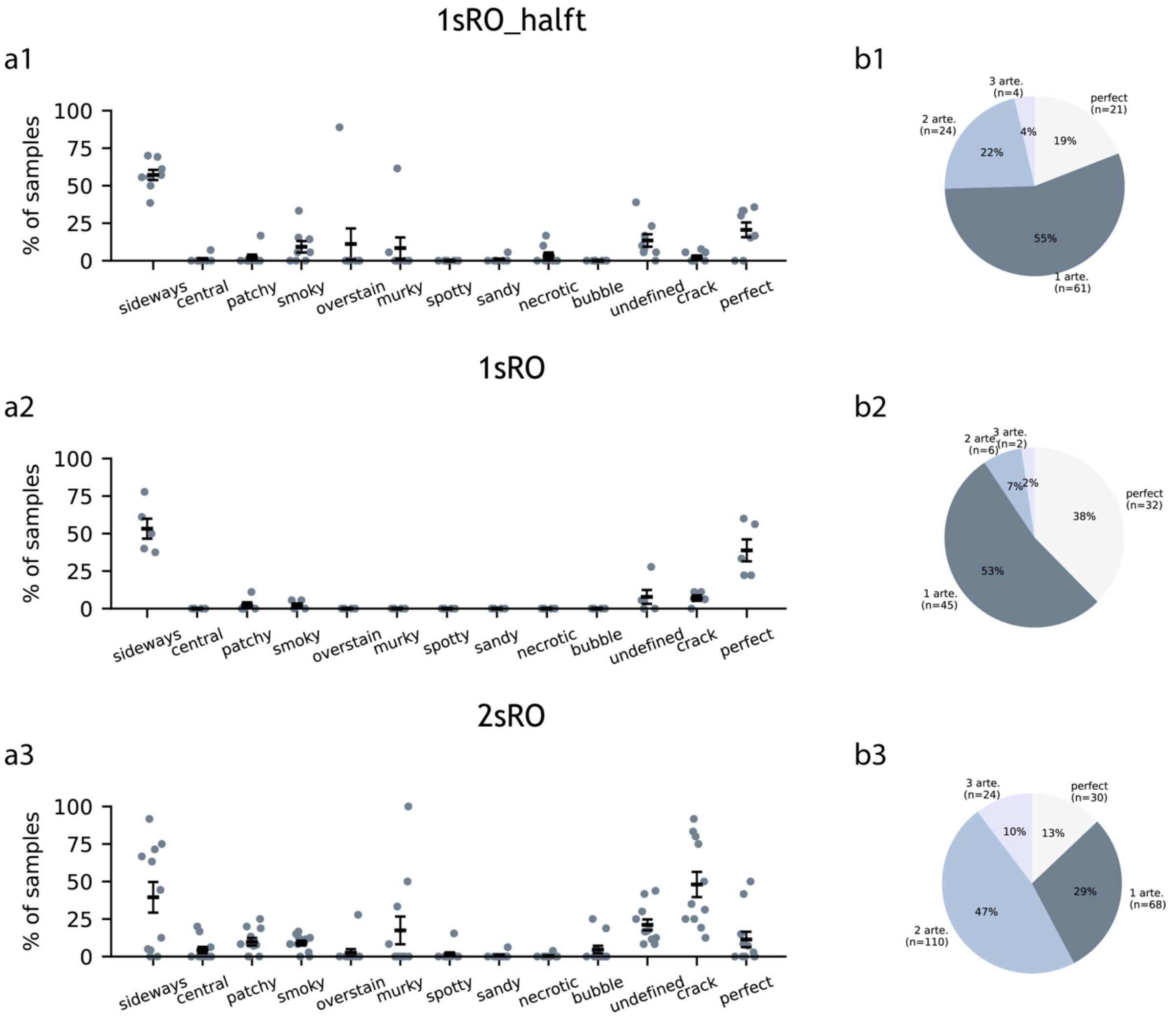

Supp. F2. Inter-batch variability of staining artefacts.

(a) Artefact incidence across multiple batches employing the same protocol. **(a1)** 1sRO_halft (n_B = 8 batches, n_S = [14, 13, 6, 10, 13, 18, 18, 18] samples in each batch, n_T is 110 samples in total). **(a2)** 1sRO (n_B = 5 batches, n_S = [16, 15, 18, 18, 18] samples per batch, n_T = 85 samples in total). **(a3)** 2sRO (n_B = 11 batches, n_S = [16, 12, 24, 20, 26, 12, 16, 12, 28, 30, 36] samples per batch, n_T = 232 samples in total). **(b)** Artefact co-occurrence in 1sRO_halft **(b1)**, 1sRO **(b2)** and 2sRO **(b3)**-based SPPs (n_B, n_S and n_T as defined in **(a)**, respectively).

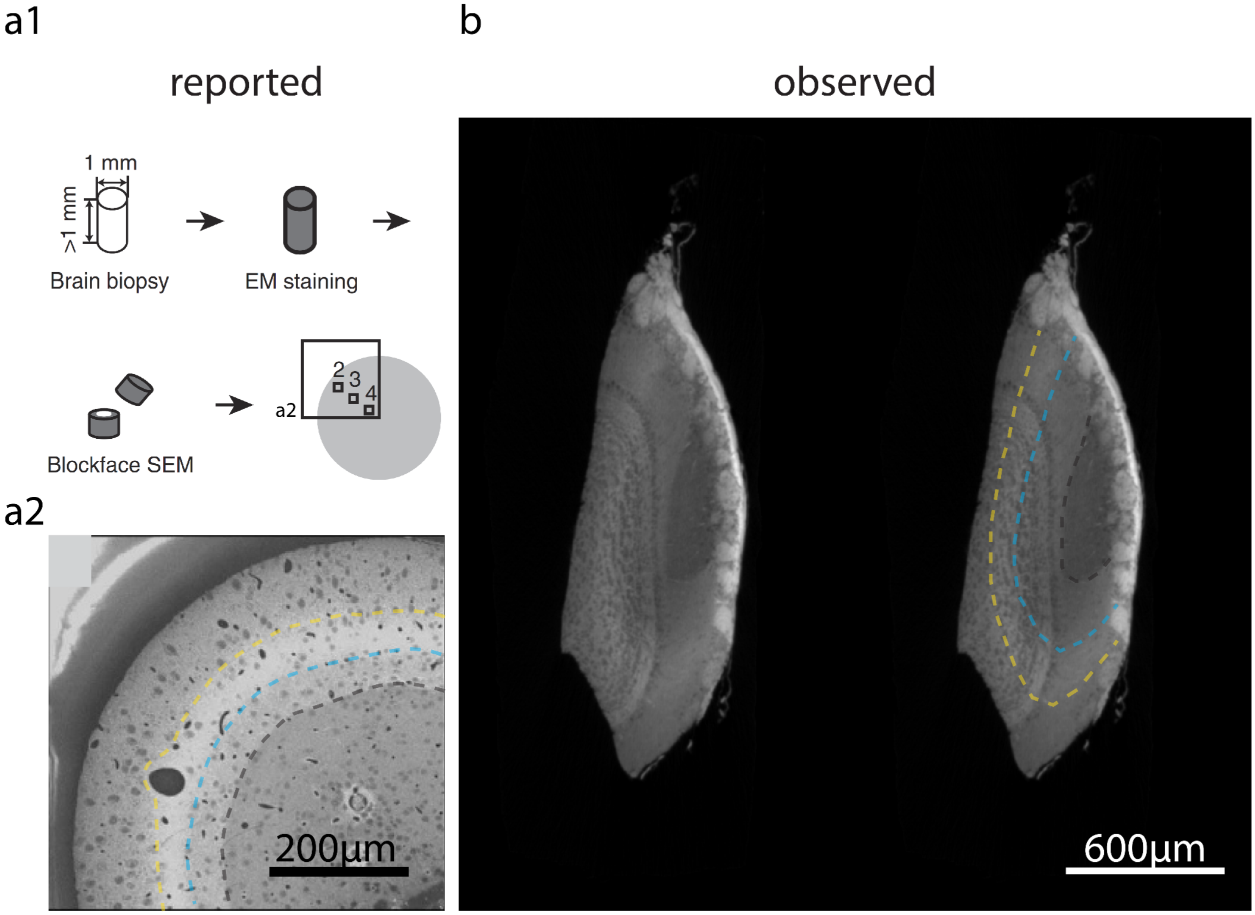

Supp. F3. Resemblance of 'sideways' to previously reported artefacts.
(a) Staining artefact previously described, displaying multiple symmetrical staining abnormalities. **(a1)** Samples were punch-dissected cylinders of adult mouse brain tissue. **(a2)** SBEM image of a cross-section of the cylinder. The boundaries of the different staining abnormalities are marked with blue, yellow and black dashed lines. **(b)** LXRT images of a first dorsal OB slice sample from this study containing 'sideways' artefact with 'compound' boundary. The boundaries of staining abnormalities equivalent to those reported in **(a2)** are delineated with blue, yellow and black dashed lines, respectively.
Panels **(a1-2)** were adapted from Hua et al. (2015).

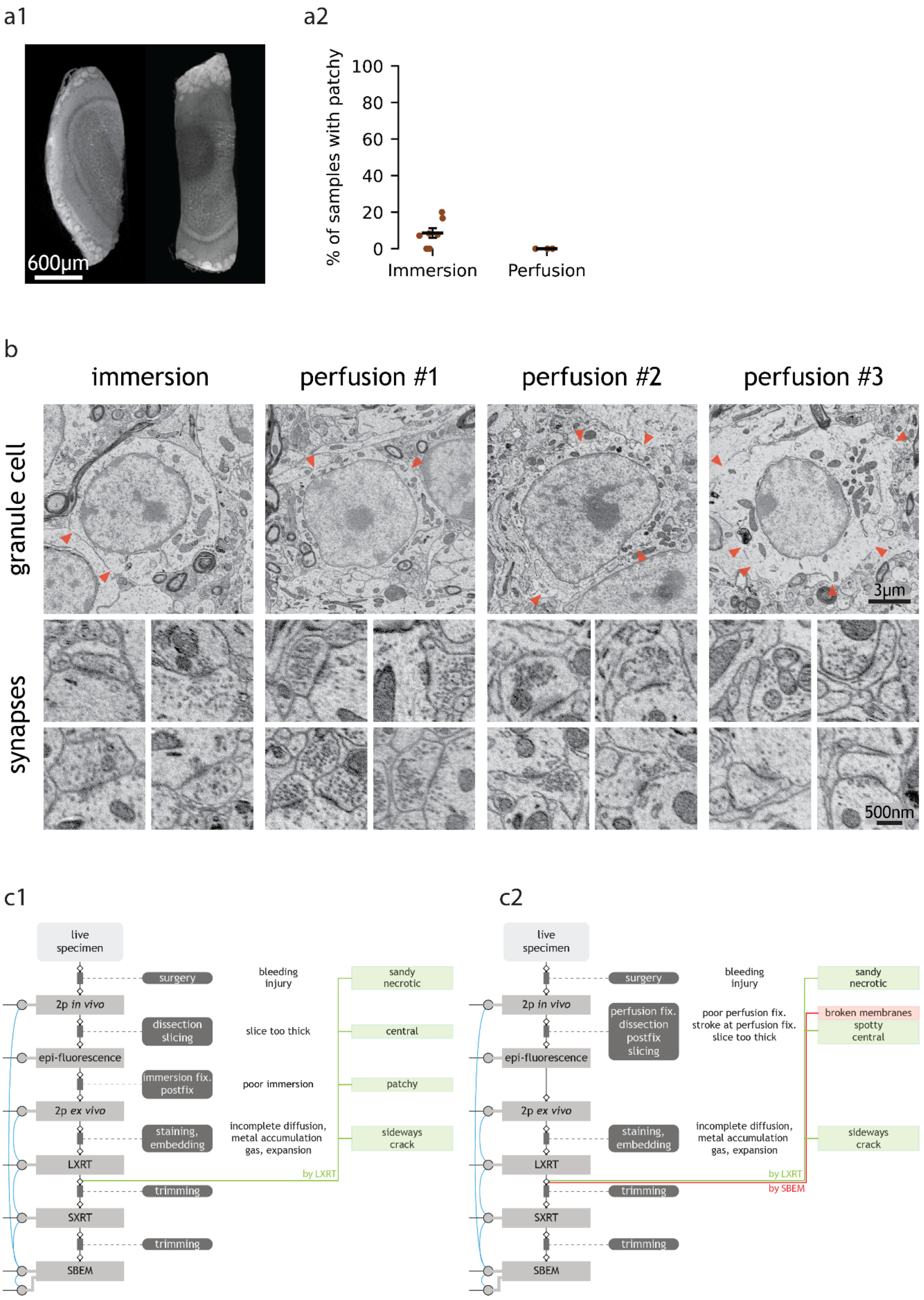
a1
600μm
a2
% of samples with patchy
100
80
60
40
20
0
Immersion
Perfusion
b
immersion
perfusion #1
perfusion #2
perfusion #3
granule cell
synapses
3μm
500nm
c1
live specimen
surgery
bleeding injury
sandy necrotic
2p in vivo
dissection slicing
slice too thick
central
epi-fluorescence
immersion fix. postfix
poor immersion
patchy
2p ex vivo
staining, embedding
incomplete diffusion, metal accumulation gas, expansion
sideways crack
LXRT
by LXRT
trimming
SXRT
trimming
SBEM
c2
live specimen
surgery
bleeding injury
sandy necrotic
2p in vivo
perfusion fix. dissection postfix slicing
poor perfusion fix. stroke at perfusion fix. slice too thick
broken membranes
spotty central
epi-fluorescence
2p ex vivo
staining, embedding
incomplete diffusion, metal accumulation gas, expansion
sideways crack
LXRT
by LXRT
by SBEM
trimming
SXRT
trimming
SBEM

Supp. F4. Workflows with different fixation methods exhibit different artefacts and require different quality control measures.

(a1) Representative LXRT images of first and second dorsal slices from an OB sample with ‘patchy’ artefact. **(a2)** Percentage of samples with ‘patchy’ in experiments fixed by immersion or perfusion. Each dot represents an experiment. Error bars show mean +/- standard error of mean. **(b)** Representative SBEM images of granule cells and synapses in the external plexiform layer of OB samples fixed by immersion or perfusion. Arrowheads point towards membrane breaks. **(c)** Structures of SPPs using immersion fixation **(c1)** and perfusion fixation **(c2)**, respectively. Black text in grey boxes states the imaging techniques; white text in boxes states the sample processing steps; diamonds represent quality control steps; grey circles represent curated datasets; blue lines represent correlations among curated datasets; artefacts are shown in colored boxes next to their hypothetical causes in line with the sample processing step that putatively generates them. Inspection of most SPP artefacts occurs after LXRT. Inspection methods include LXRT (green line) and SBEM (red line).

a

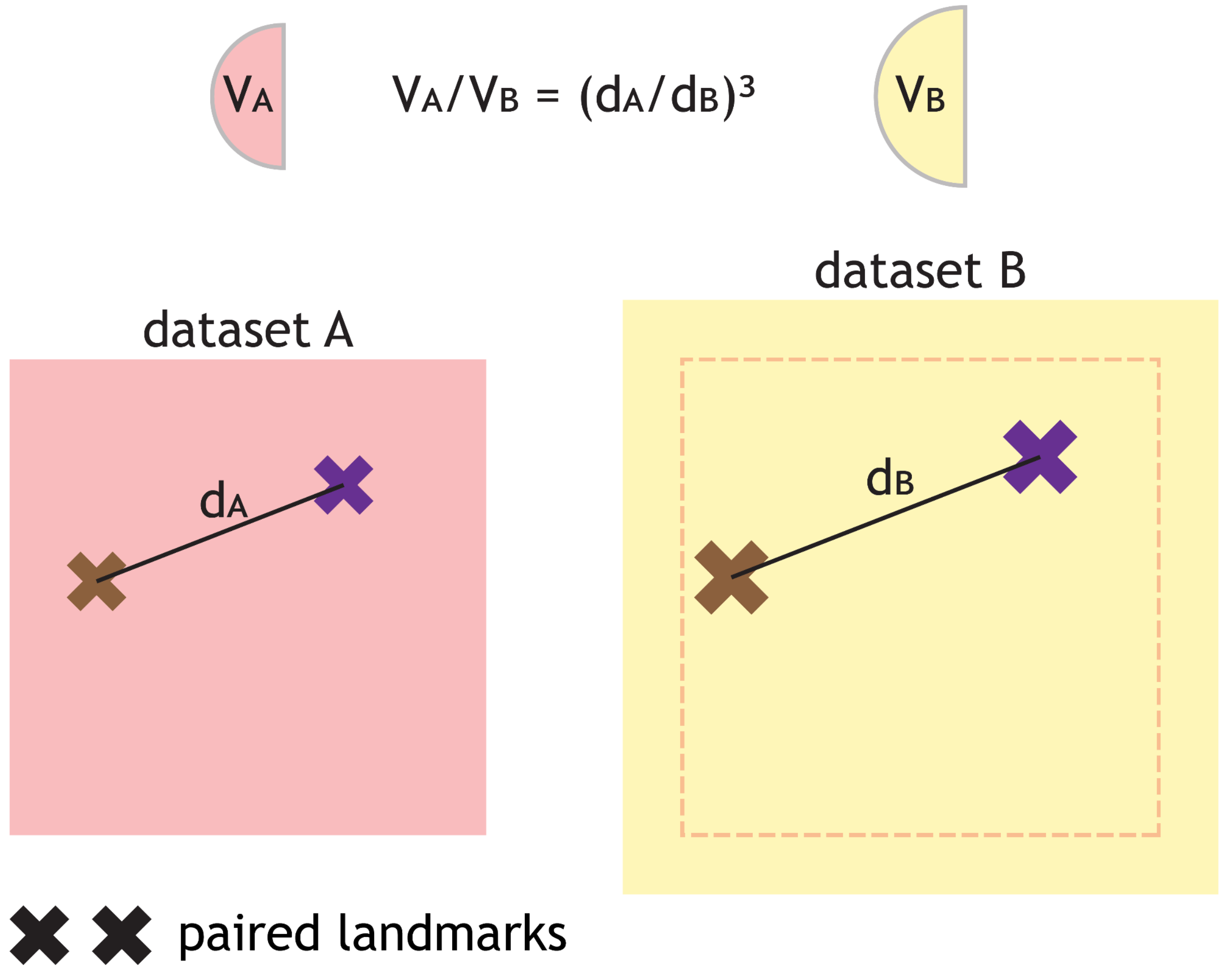

Supp. F5. Method to estimate the sample volume of fresh tissue samples.

(a) The volume ratio between two volumetric datasets A and B mapping the same specimen is estimated to be the cubed ratio of the distances between conserved pairs of landmarks in both datasets. The dashed box represents the volume of dataset A in the expanded dataset B. Symbols: *V*: volume, *d*: distance. This approach was used to estimate the fresh tissue volume of a 1st OB dorsal slice from correlated 2P and SBEM landmarks. The 2P dataset was obtained in the fresh tissue *in vivo*, whereas the SBEM dataset was acquired after staining and embedding had taken place.

Supp. Table 1. Artefact definition.
Definitions of each of the 12 artefacts (**Fig. 1c**), subtypes of 'sideways' (**Fig. 2b**) and subtypes of 'crack' (**Fig. 3a1**).

Supp. Table 2. Sample preparation protocol (SPP) details.
Summary of samples, and details on the fixation, staining, dehydration and embedding steps of all SPPs included in this study.

Supp. Table 3. Experimental and animal details of images in figures.
Animal details, experiment ID and relevant sample details (slice index, thickness, in vivo two-photon history) for every 2P, LXRT, SXRT and EM image shown in the figures.

Supp. Table 4. Mouse line details.
Founder line details of all mouse lines used in this study.

Supp. Table 5. Experimental cohort descriptions.
Sample IDs and sample selection criteria for all the analyses included in this study.

Supp. Table 6. Volume cohorts.
List of samples whose final in-resin volume was measured.

Supp. Table 7. Animals, samples, batches and experiments
Sample, batch and animal details for all plots in this study.

Supp. Table 8. Acronyms
List of acronyms used in this study.

References

Hua, Y., Laserstein, P., and Helmstaedter, M. (2015). Large-volume *en-bloc* staining for electron microscopy-based connectomics. *Nature communications* 6(1), 1-7.
